# Supplementary material for: Vagus nerve stimulation alters task‐evoked pupillary responses in older but not younger adults: A single‐blind active sham‐controlled crossover trial
Source: Alzheimers Dement. 2026 Feb 27;22(2):e71239. doi: 10.1002/alz.71239 (PMC12946818; doi:10.1002/alz.71239)
Supplement: Supplementary file 1 — Supporting Information [file ALZ-22-e71239-s002.pdf]

Supplement: Transcutaneous auricular vagus nerve stimulation administration details and participant experiences								
Participant	Age Group	Sham Amplitude	Verum Amplitude	Amplitude Difference	Time Between Conditions	First Condtion	Second Condition	Notes
149	Older	1000 μA	2100 μA	1100	35 min	Verum	Sham	Verum: Felt stimulation at 400μA and described it as a prickly sensation. As the amplitude was increased to closer to 1800μA, it continued to feel prickly. Once we hit 2100μA, participant said it was a constant vibration and was no longer prickly or annoying. Sham: Felt stimulation at 800μA and said it was comfortable closer to 1000μA.
165	Younger	1400 μA	1000 μA	-400	27 min	Verum	Sham	Both condition amplitudes were adjusted based on participant sensation.
202	Older	3400 μA	1500 μA	-1900	35 min	Sham	Verum	Sham: Felt stimulation at 3100μA and said it was prickly but became more comfortable as amplitude increased. Verum: Felt stimulation and described it as comfortable at 1500μA
206	Older	1500 μA	1200 μA	-300	40 min	Sham	Verum	Sham: Felt sensation around 1000μA and described it as prickly. It became a vibrating sensation as amplitude was increased to 1500μA. Verum: Felt the stimulation at 800μA and said it felt sharper than last time. The sensation became pinchy closer to 1400μA. We decreased the amplitude as increases seemed to make it worse. At 1200μA, it was bearable and constant vibration.
212	Older	1800 μA	1800 μA	0	35 min	Sham	Verum	Sham: First felt stimulation at 700μA and described it as feeling like "an ant crawling on [their] ear" as amplitude was increased to 1300μA, it became more uncomfortable. At 1800μA, it became a vibrating sensation that was more comfortable. Verum: Felt a small sensation at 1100μA, described it as irritating but not painful. The feeling became more of a little buzz as amplitude was increased to 1800 μA.
220	Older	900 μA	2500 μA	1600	30 min	Sham	Verum	Sham: Felt prickles at 700uA, became more constant vibration and felt comfortable at 900uA. Verum: At 1900uA, began to feel the stimulation and described it as a subtle "electrical binging" which turned into a more constant and comfortable buzzing as amplitude was increased.
222	Older	5000 μA	1400 μA	-3600	35 min	Sham	Verum	Sham: Did not feel vibration at any amplitude - took it off and tested it on fingers to ensure that it really was stimulating (it was). Verum: Felt the vibration at 900uA, said it was pin-pricky. As amplitude was increased to 1200uA, it became a more vibration-like sensation. At 1400uA, it felt comfortable and "like a small itch".

Supplement: Transcutaneous auricular vagus nerve stimulation administration details and participant experiences								
**Participant**	**Age Group**	**Sham Amplitude**	**Verum Amplitude**	**Amplitude Difference**	**Time Between Conditions**	**First Condtion**	**Second Condition**	**Notes**
235	Older	5000 µA	5000 µA	0	35 min	Verum	Sham	Verum: Did not feel stimulation at any amplitude. Sham: Did not feel stimulation at any amplitude.
239	Older	1200 µA	1400 µA	200	50 min	Verum	Sham	Verum: First felt the stimulation at 1400µA and immediately described the sensation as a slight buzz, so we did not increase the amplitude. Sham: Felt the stimulation at 1000µA and said that it was "too much", this feeling remained constant as amplitude was increased past 3000µA. It remained sharp around 3800µA, so we went down. It felt comfortable at 1200µA.
242	Older	1500 µA	1600 µA	100	35 min	Sham	Verum	Sham: Felt stimulation at 1300µA and said it was prickly, sensation became more of a constant vibration as amplitude was increased to 1500µA. Verum: Felt stimulation at 900µA and said it was prickly around 1300µA, sensation became more of a constant vibration as amplitude was increased to 1600µA.
524	Older	5000 µA	2800 µA	-2200	35 min	Sham	Verum	Sham: Did not feel stimulation at any amplitude. Verum: Felt stimulation around 2000uA and described it as prickly. Sensation became more intense as amplitude was increased and eventually became more of a constant vibration at 2800uA.
525	Older	1200 µA	1500 µA	300	35 min	Verum	Sham	Verum: Felt the stimulation at 900uA and described it as a small itch. As we increased to 1200, it began to feel more like a "mosquito biting [their] ear". This sensation turned into more of a comfortable and constant buzzing at 1500uA. Sham: Felt the stimulation at 800uA and described it as "buzzy". Sensation became more of a constant vibration closer to 1200uA.
564	Younger	900 µA	1500 µA	600	20 min	Sham	Verum	Both condition amplitudes were adjusted based on participant sensation.
571	Older	1400 µA	1800 µA	400	35 min	Verum	Sham	Both condition amplitudes were adjusted based on participant sensation.
575	Younger	1200 µA	1600 µA	400	10 min	Verum	Sham	Adjusting verum stimulation particiant reported pins and needles sensation at lower amplitudes followed by strong vibration at higher intensity.
577	Younger	1500 µA	1700 µA	200	20 min	Verum	Sham	Both condition amplitudes were adjusted based on participant sensation.
578	Younger	1100 µA	1700 µA	600	29 min	Sham	Verum	Both condition amplitudes were adjusted based on participant sensation.

Supplement: Transcutaneous auricular vagus nerve stimulation administration details and participant experiences								
**Participant**	**Age Group**	**Sham Amplitude**	**Verum Amplitude**	**Amplitude Difference**	**Time Between Conditions**	**First Condtion**	**Second Condition**	**Notes**
593	Younger	800 µA	1000 µA	200	24 min	Verum	Sham	Felt a sensation in both conditions very early. Adjustment to vibration without pain.
595	Younger	900 µA	1200 µA	300	20 min	Verum	Sham	Verum: Slight stinging sensation at low intensity, increasing intensity produced dominant vibration sensation. Sham: tingling sensation reported increased to slight vibration.
596	Older	1600 µA	2400 µA	800	55 min	Sham	Verum	Both condition amplitudes were adjusted based on participant sensation.
597	Younger	1400 µA	1400 µA	0	40 min	Verum	Sham	"Electrical sensation" in both conditions; participant did not want a higher intensity.
600	Older	3000 µA	3000 µA	0	3.5 hrs	Sham	Verum	Verum: participant did not seem to feel much of anything; settled at 3000 µA. Sham: No sensation at 3000.
608	Older	2600 µA	3200 µA	600	40 min	Sham	Verum	Sham: Consistently felt a tingling/vibrating sensation that peaked at noted amplitude. Verum: worked our way up to a constant vibration, had to readjust amplitude a few times to make sure the vibration was felt.
609	Younger	1700 µA	1500 µA	-200	25 min	Verum	Sham	Verum: Moderately painful itching sensation as intensity increased. The pain did not go away with higher intensities–decreased to 1500µA to avoid. Sham: Mild sensation with little to no pain at 1700.
610	Younger	2000 µA	2100 µA	100	26 min	Sham	Verum	Sham: Sharp sensation which quickly subisded to vibrating sensation after a couple seconds of stimulation. Verum: tingling/vibrating sensation without pain.
617	Younger	3200 µA	3100 µA	-100	30 min	Verum	Sham	Verum: reported prickling sensation at lower amplitudes "as if bug is buzzing in my ear." At higher amplitudes, reported lower (but still present) levels of this feeling. Sham: reported prickling sensation at lower amplitudes, felt tickling sensation on earlobe that subsided as we increased the amplitude.
619	Younger	3300 µA	3000 µA	-300	30 min	Verum	Sham	Verum: Felt no sensation until 1300µA, and then reported it as painful when above 3000µA, so we kept it at 3000µA. Sham: Felt more sensation in sham than verum position. As amplitude increased, prickling sensation became replaced with less painful buzzing sensation. Left intensity at 3300µA.

Supplement: Transcutaneous auricular vagus nerve stimulation administration details and participant experiences								
**Participant**	**Age Group**	**Sham Amplitude**	**Verum Amplitude**	**Amplitude Difference**	**Time Between Conditions**	**First Condtion**	**Second Condition**	**Notes**
621	Younger	1000 µA	900 µA	-100	20 min	Verum	Sham	Verum: Very sensitive ear, too painful at any amplitude above 900µA. Tried going up to find that "buzzing" sensation, but there was no level above 900µA that was not painful. Left it at 900µA. Sham: Again, very sensitive. Too painful at any amplitude above 1000µA. Left it at 1000µA.
624	Younger	2600 µA	2800 µA	200	38 min	Sham	Verum	Sham: At lower amplitudes, described a pricking sensation. At higher amplitudes, described a buzzing sensation. Verum: Went typically/smoothly.
625	Older	2300 µA	2600 µA	300	45 min	Verum	Sham	Verum: Pretty sensitive to the device, still felt a little painful but tolerable even at high amplitudes. Sham: Ear was also pretty sensitive at all amplitudes.
627	Younger	2800 µA	2400 µA	-400	35 min	Verum	Sham	Verum: "Electrical" sensation. Sham: similar sensations, no issues.
628	Younger	1700 µA	1900 µA	200	32 min	Sham	Verum	Sham: vibrating, massage sensation at around 1200µA. Verum: described very similar sensations to sham.
629	Younger	2300 µA	2600 µA	300	30 min	Verum	Sham	Verum: Reported never feeling the pinprick sensation and immediately feeling the buzzing sensation. Sham: No issues, reporting pinprick sensation that turned into buzzing sensation.
635	Older	5000 µA	3800 µA	-1200	30 min	Verum	Sham	Verum: Did not feel anything until 3600µA, at which point it felt prickly, felt more of a constant tingling as we increased amplitude Sham: Did not feel anything, maxed out at 5000µA
636	Older	1000 µA	2300 µA	1300	35 min	Sham	Verum	Sham: Felt stimulation at 900uA and described it as a low vibration which became more pronounced as we increased to 1200uA. We selected 1000uA for comfort. Verum: Felt a small tingling sensation at 2000uA which became more comfortable with a slight increase in intensity.
637	Older	1400 µA	1100 µA	-300	45 min	Verum	Sham	Verum: Felt stimulation at 1100uA and described it as a warm tingling that was comfortable, also said it felt "hot". Sham: Felt stimulation at 1200uA and described it as a little tingling sensation - became more comfortable with slight increase.

Supplement: Transcutaneous auricular vagus nerve stimulation administration details and participant experiences								
**Participant**	**Age Group**	**Sham Amplitude**	**Verum Amplitude**	**Amplitude Difference**	**Time Between Conditions**	**First Condtion**	**Second Condition**	**Notes**
638	Older	1000 μA	1000 μA	0	35 min	Sham	Verum	Sham: Felt stimulation at 800uA and described it as a small ache on his earlobe - became more intense but more constant and bearable at 1000uA. Verum: Felt stimulation at 800uA and described it as a pin-like sensation. Increasing stimulation brought it to more of a comfortable buzzing sensation. Once we got to 1200uA, it became more uncomfortable so we went back down. 1000uA was the happy point - comfortable enough for 8 minutes but strong enough to feel.
640	Younger	800 μA	500 μA	-300	30 min	Sham	Verum	Sham: Felt stimulation at 800uA and described it as a buzzing sensation. Increasing stimulation to 1000uA made it more uncomfortable so we went back down to 800uA. Verum: Felt stimulation at 500uA and described it as a prickly sensation. Increasing stimulation to 800uA made it more uncomfortable so we went back down to 500uA.
642	Younger	2200 μA	3000 μA	800	30 min	Sham	Verum	Sham: Felt stimulation at 800uA and described it as prickly. Increasing the amplitude made it more of a comfortable buzzing sensation. Verum: Felt stimulation at 800uA and described it as prickly. Increasing the amplitude made it more of a comfortable buzzing sensation, at 3500uA they could not feel anything so we went back down a little.
644	Younger	1200 μA	300 μA	-900	35 min	Sham	Verum	Sham: Felt stimulation at 700uA and described it as "itchy". At 1000uA, it became more of a vibration, but also felt like an ant crawling on their ear. At 1200, it became more comfortable. Verum: Felt stimulation almost immediately. Increasing to 500uA made it uncomfortably stingy and the sensation got worse with further increases so we reduced the amplitude.
646	Younger	1500 μA	2400 μA	900	35 min	Sham	Verum	Sham: Felt stimulation at 1000uA and didn't feel any discomfort. As the amplitude was increased, the comfortable vibration-like sensation persisted. Verum: Felt stimulation at 1000uA and described it as prickly. As the amplitude was increased to 1500uA, a more comfortable vibration-like sensation began to emerge, which then became dominant with further increases.
